# Non-structural carbohydrates in maize with different nitrogen tolerance are affected by nitrogen addition

**DOI:** 10.1371/journal.pone.0225753

**Published:** 2019-12-05

**Authors:** Yawei Wu, Bo Zhao, Qiang Li, Fanlei Kong, Lunjing Du, Fang Zhou, Haichun Shi, Yongpei Ke, Qinlin Liu, Dongju Feng, Jichao Yuan

**Affiliations:** 1 Key Laboratory of Crop Ecophysiology and Farming System in Southwest China, Ministry of Agriculture/College of Agriculture, Sichuan Agricultural University, Chengdu, P.R. China; 2 Chongqing Key Laboratory of Economic Plant Biotechnology/Collaborative Innovation Center of Special Plant Industry in Chongqing/Institute of Special Plants, Chongqing University of Arts and Sciences, Chongqing, P.R. China; 3 Crop Ecophysiology and Cultivation Key Laboratory of Sichuan Province, Chengdu, P.R. China; Tennessee State University, UNITED STATES

## Abstract

Non-structural carbohydrates (NSCs) are an important energy source for plant growth and metabolism. Analysis of NSC changes can provide important clues to reveal the adaptation mechanisms of plants to a specific environment. Although considerable differences have been reported in NSCs in response to nitrogen (N) application among crop species and cultivars, previous studies have mostly focused on the differences in leaves and stems. However, the effects of N on the characteristics of accumulation and translocation of NSC in maize with different levels of N tolerance remain unclear. To determine differences in the N levels, two cultivars (N-efficient ZH311 and N-inefficient XY508) were grown in field pots (Experiment I) and as hydroponic cultures (Experiment II) and were supplemented with different concentrations of N fertilizer. In both experiments, low-N stress significantly increased the accumulation of NSCs in maize vegetative organs and increased the translocation rate of NSCs in the stems and their apparent contribution to yield, thereby reducing the yield loss caused by low-N stress. N application had a greater effect on starch content in the vegetative organs of ZH311, but had less effect on soluble sugar (SS) and NSC content in the whole plant and starch content in the ears. ZH311 could convert more starch into SS under low N conditions to adapt to low N environments than XY508, while ensuring that grain yield and starch quantity were not affected. This is evidently an important physiological mechanism involved in this cultivar’s tolerance to low N conditions.

## Introduction

Nitrogen (N) exerts a strong influence on the growth and development of plants under different environmental conditions. Application of N through chemical fertilizers is the dominant source of N input in crop production systems [1]. However, excessive application of N fertilizer negatively influences both crop yield and the environment by increasing N-related emissions [2–3]. This remains a problem for agricultural production in China [4]. The N-use efficiency of cereals is only about 33% in China [5]. There is a growing conflict between increasing food demand and limited water and fertilizer resources; therefore, increasing crop yield and N-use efficiency is a global challenge [3].

Maize (*Zea mays* L.) is one of the most important crops globally. In China, maize production was 216 million tons in 2014, which was more than rice (206 million tons) and wheat (126 million tons) [6]. Nevertheless, the amount of maize produced per cultivated area in China is only about 5900 kg·hm^-2^ in 2012–2014, which is considerably below the level of 9500 kg·hm^-2^ per area in America [7]. This suggests that maize yield needs to be further improved in China. Increases in maize yield rely on increased N input, but this can also result in higher N loss and the associated increase in environmental pollution [8–9]. Only yield increase driven by improved N-use efficiency will provide the necessary maize production levels without increasing N emission. Reducing the fertilizer requirement of maize by breeding and using N-efficient cultivars with improved nutrient uptake and use is an important goal for agriculture [10–11].

Carbohydrate is the main product of plant photosynthesis and can be divided into structural carbohydrates and non-structural carbohydrates (NSCs) [12]. Structural carbohydrates are mainly used for the production of lignin and cellulose by plants. Non-structural carbohydrates, mainly glucose and starch [13], play important roles in the metabolic processes of plants. The level of NSCs in plants can not only reflect their ecophysiological and growth status, but also the plant’s response to external stress [13]. Ainsworth and Bush [14] proposed that improving the output and reuse capability of NSCs is important to achieve high crop yield under the background of climate change. Several factors may influence plant NSC content, and N level is one of them. On one hand, N addition could improve plant photosynthetic rate, which could increase plant NSC [15]. On the other hand, N addition may also damage the photosynthetic system due to N assimilation, and could decrease plant NSCs [16]. Several studies have reported the response of plant NSCs to N addition. Pan [17] found that N application affected the accumulation of NSC in the stem of rice. Under low N conditions, the concentration of NSCs and apparent transferred mass of NSCs were higher than those under high N conditions. Overuse of N fertilizer is not conducive for the accumulation and translocation of photosynthates. Peng et al. [18] observed that N treatment strongly negatively influenced ear and leaf NSC concentration in maize, and that ear and leaf carbohydrates accumulated in plants grown under low N conditions. Improving the accumulation of ear and leaf carbohydrates may increase maize yield under low N conditions [19]. Variation in NSC content in response to applied N has also been studied in different plant species and cultivars. Xiong et al. [20] suggested that variations in high-temperature tolerance among rice cultivars and N treatments are related to plant NSC concentration. They found that the NSC concentrations in high-temperature-tolerant cultivars were higher than those in high-temperature-sensitive cultivars under any N levels; however, the underlying mechanism is not fully understood.

Although considerable differences in NSC response to N application among crop species and cultivars have been reported, most research has focused on differences in the accumulation and translocation in the leaves and stems at the panicle stage. Therefore, the effects of N on NSCs in maize cultivars with different levels of N tolerance remain unclear hindering our understanding of the availability of carbohydrates for plant growth and its relationship with N. In our study, a field experiment and hydroponic experiment with N-efficient and N-inefficient cultivars, which were selected according to the results of a preliminary screening experiment, were carried out under different N levels. The objectives of the study were to: (1) clarify the differences in NSC content in different organs of maize at different growth stages under different nitrogen levels; (2) study the differences in NSCs between N-efficient and N-inefficient maize cultivars; and (3) study the effects of NSCs on low-N tolerance of N-efficient cultivars and its mechanisms.

## Materials and methods

### Ethics statement

No specific permissions were required for the described field studies, and the field studies did not involve endangered or protected species. All experiments were performed according to the institutional guidelines of the Sichuan Agricultural University, China.

### Experimental condition

This study comprised two experiments conducted in two different regions over a span of 4 years. Experiment I (Exp. I) was a field pot experiment conducted in Jianyang, Sichuan Province, China (30°38′N, 104°53′E), during the 2014 and 2015 growing seasons. Experiment II (Exp. II) was a hydroponic culture experiment set in Wenjiang, Sichuan Province, China (30°71′N, 103°87′E), during 2016 and 2017. The material for Exp. I was grown under local natural cultivation conditions, while that for Exp. II was grown under artificial controlled growth conditions (i.e., artificial incubation). The experimental soils for Exp. I were collected from an area of long-term maize production at Jianyang. The properties of the tested soil prior to the start of the pot experiment are shown in Table 1. Jianyang has a subtropical monsoon climate with annual average precipitation of 874 mm, annual average temperature of 17°C, and annual frost-free period of 311 d.

**Table 1:** Properties of the tested soil in the experiment sites in 2014–2015.

Year	Organic matter (g·kg^-1^)	Total N (g·kg^-1^)	Total P (g·kg^-1^)	Total K (g·kg^-1^)	Alkali hydrolysable N (mg·kg^-1^)	Olsen-P (mg·kg^-1^)	Exchangeable K (mg·kg^-1^)	pH
2014	15.75	1.75	0.57	12.61	39.26	2.55	139.33	7.59
2015	13.30	1.56	0.40	8.25	36.34	2.27	128.50	8.16

### Experimental design

In Exp. I, a randomized block design with three replicates was used. The two cultivars (ZH 311, N-efficient cultivar and XY508, N-inefficient cultivar) [21] and three N rates in 2014 (B0, 0 kg·ha^-1^; B1, 150 kg·ha^-1^; and B2, 300 kg·ha^-1^) and four N rates in 2015 (B0, 0 kg·ha^-1^; B1, 150 kg·ha^-1^; B2, 300 kg·ha^-1^; and B3, 450 kg·ha^-1^) were randomly assigned to each replicate. Among them, the B0 treatment involved severe low-N stress and the B1 treatment involved mild low-N stress. The experiment was carried out in plastic pots with a mean diameter of 30 cm and height of 30 cm. Each replicate consisted of 20 pots, and there were 360 pots in 2014 and 480 pots in 2015. Each pot was filled with 20 kg of soil and arranged in alternating wide and narrow rows (1.4 m + 0.4 m) equivalent to a maize density of 52500 plants ha^-1^. The seeds were germinated in a nursery. Following this, the seedlings with at least two fully expanded leaves were transplanted at a density of two seedlings per pot. All pots were treated with 72 kg·ha^-1^ of P_2_O_5_ as a single superphosphate fertilizer and 90 kg·ha^-1^ of K_2_O in the form of potassium chloride as a basal fertilizer. N fertilizer in the form of urea was equally split applied as a basal fertilizer and supplementary fertilizer, and the supplementary fertilizer was applied at the V12 stage. All other aspects of management were identical for each plot throughout the experimental period.

Exp. II involved a completely randomized block design with two maize cultivars (ZH311 and XY508) and two N treatments (N0, 0.04 mM and N2, 4 mM) in 2016 and three N treatments (N0, 0.04 mM; N1, 0.4 mM; and N2, 4 mM) in 2017. For sterilization, maize seeds were soaked in 10% (v/v) H_2_O_2_ for 40 min, and then washed several times with distilled water [22]. Thereafter, the seeds were germinated in vermiculite at 28°C under a 14/10 h light:dark photoperiod at the light intensity of 300 μmol·m^-2^·s^-1^. When two leaves were fully expanded, the endosperm was removed to N initiate, and uniform seedlings were transferred into black plastic pots (length 50 cm × width 35 cm × height 18 cm, 20 seedlings per pot) containing 10 L of nutrient solution. Ca(NO_3_)_2_ was used as a N source, and Ca^2+^ deficiency was rectified by supplementing CaCl_2_ under low-N treatments. The basic nutrient solution contained 0.75 mM K_2_SO_4_, 0.1 mM KCl, 0.25 mM KH_2_PO_4_, 0.65 mM MgSO_4_, 0.13 mM EDTA-Fe, 1.0 mM MnSO_4_, 1.0 mM ZnSO_4_, 0.1 mM CuSO_4_, and 0.005 mM (NH4)_6_Mo_7_O_24_. The seedlings were grown in a growth chamber at 28°C/22°C under a 14/10 h light:dark cycle. The nutrient solution was replaced every third day and aerated continuously using an electric pump. The pH was adjusted to 6.0 with KOH.

### Sampling and measurements

In Exp. I, samples were collected from two neighboring pots (four plants) at different stages: jointing (V6), big trumpet (V12), silking (R1), milk-ripe (R3), and maturity (R5). Each sample was divided into root, leaf lamina, stem plus sheath, and panicle (grain and bract plus cob at R5). In Exp. II, at 9 and 12 days after N treatment, eight uniform seedlings were sampled from each treatment (with three replicates), and each sample was divided into aboveground (leaves and stems) and underground (roots). Fresh samples were oven-dried at 105°C for 60 min to stop the enzymatic reaction in plants and prevent the conversion and consumption of chemicals [7], and then at 80°C until a constant weight; each sample was milled to a fine powder until the material passed through a 0.25-mm screen.

NSC concentrations were determined according to the method described by Xiong et al. [20]. Approximately 0.5 g of each dried powder sample was added to 10 mL of distilled water and placed in a water bath at 100°C for 2 h. Powdered samples were solubilized using 80% ethanol in an 80°C water bath and the extraction process was repeated three times to ensure all soluble sugars (SSs) from the samples were collected. The final extract was centrifuged for 15 min at 13 500 × *g*. Starch, the insoluble sugar remaining in the pellet after removal of the supernatant, was solubilized following the procedure of Jung and Burd [12]. Subsequently, 1.5 mL of this reaction solution was transferred to a cuvette, and then 3.0 mL of distilled water, 1.0 mL of anthrone reagent, and 5.0 mL of H_2_SO_4_ were added. The cuvettes were then heated in a boiling water bath for 2 min. Finally, the absorbance of each solution at 630 nm was measured. NSCs were defined as the sum of SS and starch concentrations. The apparent transferred mass (ATM_NSC_), apparent ratio transferred (AR_NSC_), and apparent contribution of transferred of NSC (AC_NSC_) were calculated according to Pan [17]:

$${\mathrm{ATM}}_{\mathrm{NSC}}=total\;mass\;of\;NSC\;in\;stem\;at\;R1–total\;mass\;of\;NSC\;in\;stem\;at\;R5$$

$${\mathrm{AR}}_{\mathrm{NSC}}={ATM}_{\mathrm{NSC}}/total\;mass\;of\;NSC\;in\;stem\;at\;R1\times 100$$

$$AC_{\mathrm{NSC}}={\mathrm{ATM}}_{\mathrm{NSC}}/yield\;per\;panicle\times 100$$

### Statistical analysis

Significant differences between the mean values were tested using the one-way ANOVA with the least significant difference (LSD) test at 0.05 level of probability with SPSS 20.0 statistical software (SPSS Inc., Chicago, IL, USA). The graphs were prepared using Origin Pro 9.0 (Origin Lab Inc., Hampton, VA, USA).

## Results

### Dry weight

**Fig 1:**
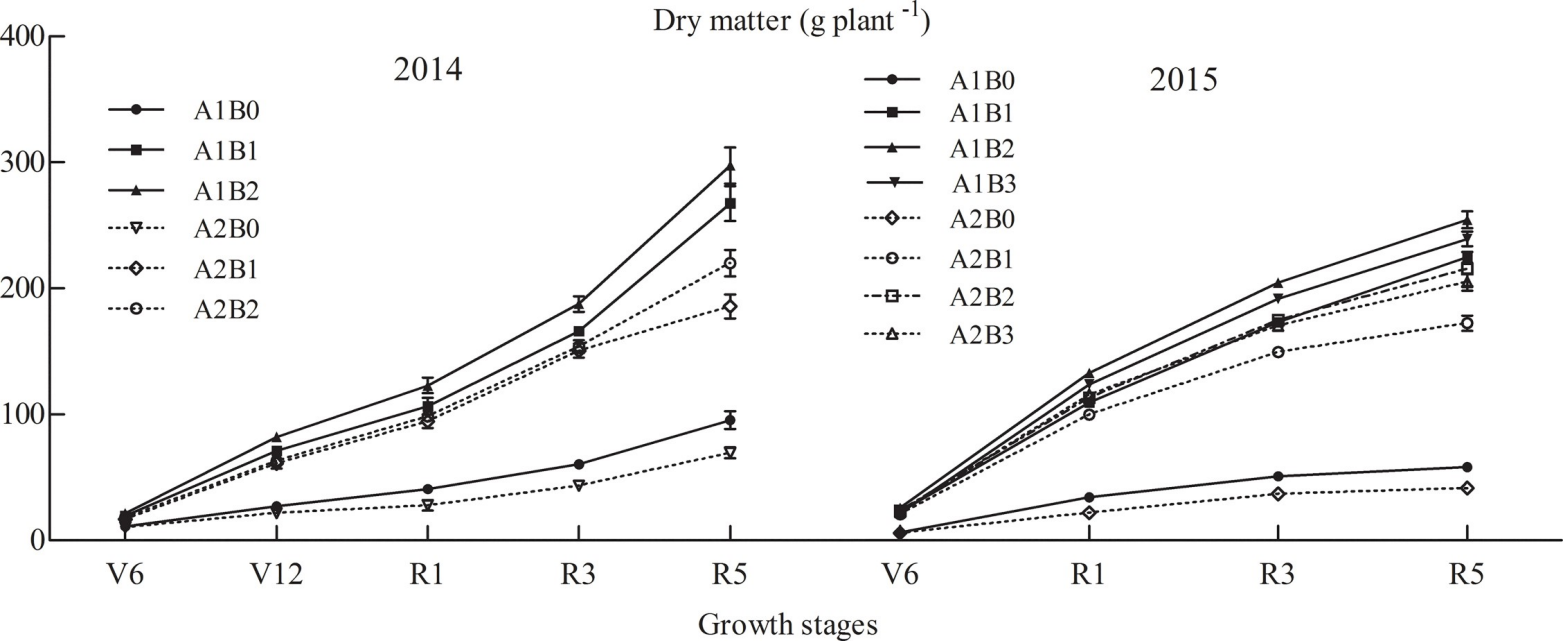
Dry weight per plant in different growth periods of maize under various treatments. Note: V6, jointing (V6); V12, big trumpet; R1, silking (R1); R3, milk-ripe; and R5, maturity. A stands for cultivars: A1, ZH311; A2, XY508. B stands for N treatments: B0, 0 kg·ha^-1^; B1, 150 kg·ha^-1^; B2, 300 kg·ha^-1^; and B3, 450 kg·ha^-1^.The point in R5 indicates soluble sugar content in grains.

There was a significant difference in biomass between the two maize cultivars in Exp. I (Fig 1). The dry weight of ZH 311 at V6, V12 (except 2015), R1, R3 and R5 were 16.46%, 23.83%, 30.59%, 29.86% and 38.94% higher than that of XY 508 in 2014 respectively, and in 2015, they were 4.17%, 14.13%, 16.62% and 22.48% higher respectively. Compared with treatment of 0 kg·ha^−1^, N treatments (150–450 kg·ha^−1^) averaged the dry weight of Z H 311 increased by 175.48% in 2014 and by 312.20% in 2015, XY 508 increased by 192.03 and 378.01%, respectively.

**Fig 2:**
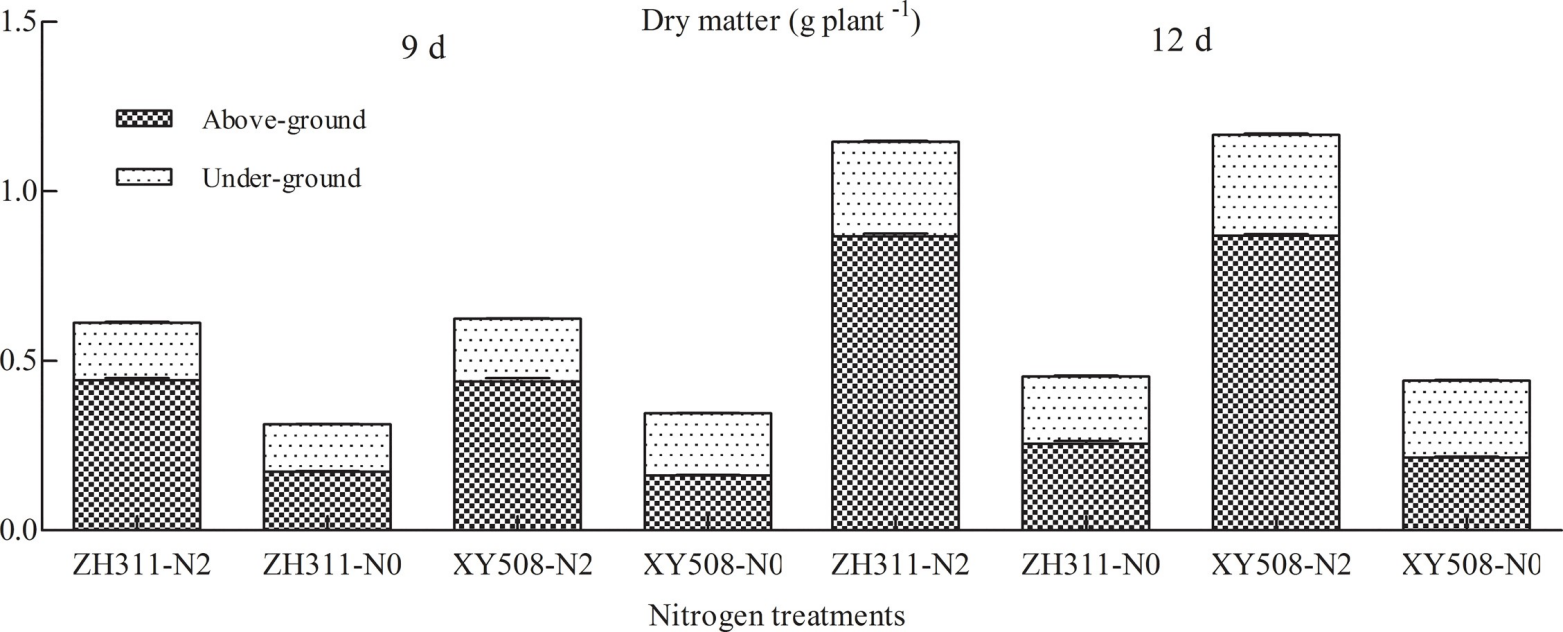
Dry weight per plant of maize in the seedling stage under different nitrogen treatments (2016). Note: N0, 0.04 mM; N2, 4 mM.

In Exp. II (Fig 2), compared with 4 mM treatment, the dry weight in ZH311 of under-ground in treatment of 0.04 mM decreased by 18.24% and 9.91% respectively at 9 and 12 d; the above-ground decreased by 60.89% and 63.08%; and the dry matter per plant decreased by 49.02% and 44.70%. The changes of XY 508 were 28.90%, 23.82%, 70.56%, 75.24%, 60.43% and 62.10%, respectively.

### Soluble sugar content

**Fig 3:**
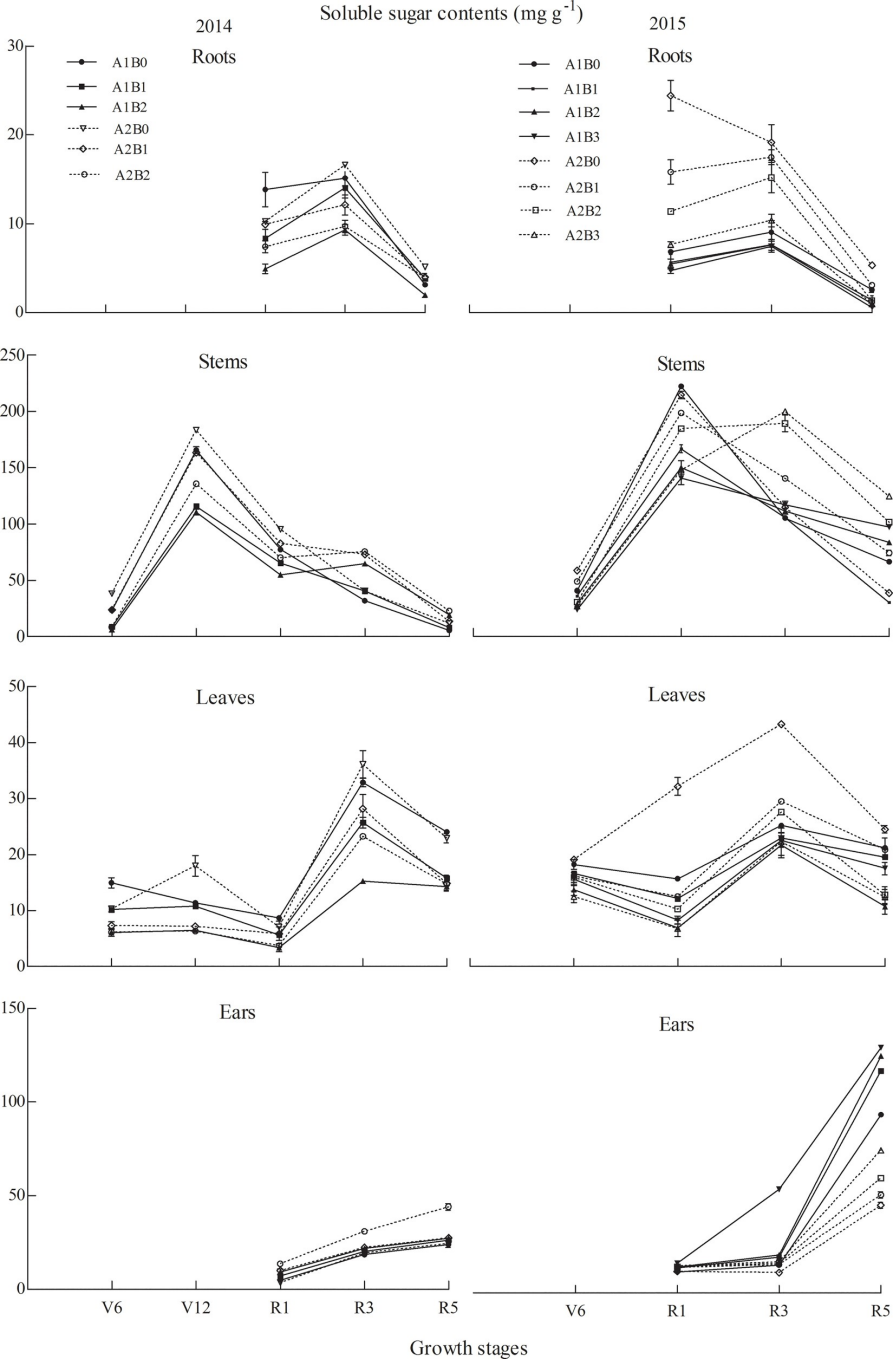
Soluble sugar content in different growth periods of maize under various treatments. Note: V6, jointing (V6); V12, big trumpet; R1, silking (R1); R3, milk-ripe; and R5, maturity. A stands for cultivars: A1, ZH311; A2, XY508. B stands for N treatments: B0, 0 kg·ha^-1^; B1, 150 kg·ha^-1^; B2, 300 kg·ha^-1^; and B3, 450 kg·ha^-1^.The point in R5 indicates soluble sugar content in grains.

**Fig 4:**
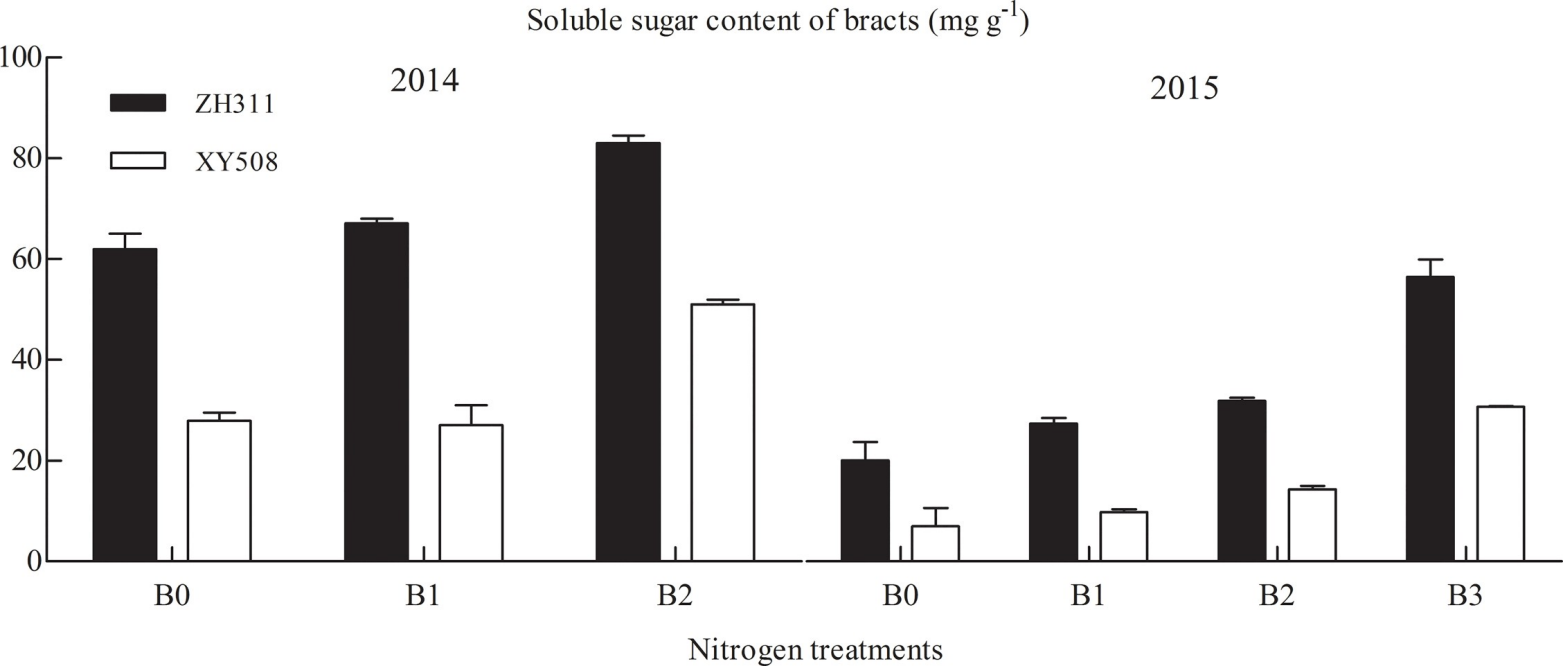
Soluble sugar content in the bracts at maturity under different nitrogen treatments. Note: B0, 0 kg·ha^-1^; B1, 150 kg·ha^-1^; B2, 300 kg·ha^-1^, and B3, 450 kg·ha^-1^.

In Exp. I (Figs 3 and 4), SS in ZH311 roots under the 150 and 300 kg·ha^-1^ treatments decreased by 28.44% in two years compared with that under the 0 kg·ha^-1^ treatment, and then decreased by 41.95% in 2015 under 450 kg·ha^-1^ treatment. In XY508, SS decreased in the 150 and 300 kg·ha^-1^ treatments with an average of 31.71% for two years, and in the 2015 450 kg·ha^-1^ treatment, it decreased by 65.31%. In the leaves, throughout the growth period of ZH311, the SS decreased by 31.24% under the 150 and 300 kg·ha^-1^ treatments compared with that under the 0 kg·ha^-1^ treatment, and decreased by 22.64% in 2015. The SS of XY508 under the 150 and 300 kg·ha^-1^ treatments decreased by 37.58% compared with that under the 0 kg·ha^-1^ treatment, and that under the 450 kg·ha^-1^ treatment decrease by 49.54%. During the V6 to R1 stages, the SS of ZH311 under the 0–450 kg·ha^-1^ treatments decreased by 35.10%. During the R1 to R5 stages, it increased by 17.88%, and the 300 kg·ha^-1^ treatment had the highest effect. The SS of XY508 under the 0–450 kg·ha^-1^ treatments during the V6 to R1 stages decreased by an average of 30.22% and increased by 42.31% during the R1 to R5 stages. The 450 kg·ha^-1^ treatment had the highest effect. At the R1 stage, the SS in the stem of ZH311 decreased by 20.40% (150 kg·ha^-1^) and 61.77% (300 kg·ha^-1^), compared with that in the stem of ZH311 under the 0 kg·ha^-1^ treatment for two years, and by 36.74% under the 450 kg·ha^-1^ treatment in 2015. The SS in XY508 under the 150–300 kg·ha^-1^ treatments decreased by 10.26% (150 kg·ha^-1^) and 20.18% (300 kg·ha^-1^), and under the 450 kg·ha^-1^ treatment, it decreased by 31.18% in 2015. At the R1 stage, under the 0 kg·ha^-1^ treatment, the SS in the stem of ZH311 was higher than that of XY508. During the R1 and R3 stages, the SS in the ears of ZH311 increased by 28.61% compared with that under the 0 kg·ha^-1^ treatment averaged for two years under the 150–450 kg·ha^-1^ treatments. At R5, the SS content in the grain increased by 18.61% and that of bract increased by 32.42%. During the R1 and R3 stages, in XY508 under the 150–450 kg·ha^-1^ treatments, the SS content increased by 33.36%; at R5, the SS content in the grain increased by 25.71% and that in the bract increased by 41.68%.

In Exp. II (Table 2), SS in the aboveground material of ZH311 in 2016 and 2017 decreased on an average by 51.56% (0.4 mM) and 68.83% (4 mM) compared with that under 0.04 mM, and that in the underground material decreased by 28.91% (0.4 mM) and 49.13% (4 mM) at 9 d after treatment. At 12 d, SS in the aboveground material decreased by 62.44% (0.4 mM) and 71.82% (4 mM), and that in the underground material decreased by 13.44% (0.4 mM) and 36.41% (4 mM). The SS content in XY508 at 9 d after treatment in the aboveground material under 0.4 and 4 mM treatments decreased by 60.79% and 62.57%, respectively, and that in the underground material decreased by 18.85% and 41.17%, respectively. At 12 d, the SS content in the aboveground material decreased by 62.12% and 67.40%, respectively, and that in the underground material decreased by 36.08% and 39.10%, respectively. It is noteworthy that SSs in the roots were observed in Exp II but were not detected at the V6 and V12 stages in Exp. I.

**Table 2:** Soluble sugar content in maize at the seedling stage under different nitrogen treatments (mg g^-1^).

Cultivar	Treatment	Above-ground		Under-ground	
		2016	2017	2016	2017
9 d after N treatments					
ZH311	N0	26.30 ± 1.26 a	63.19 ± 1.82 a	11.21 ± 0.96 a	28.82 ± 0.75 b
	N1	-	30.61 ± 0.32 b	-	20.49 ± 0.74 d
	N2	7.60 ± 0.67 c	21.13 ± 0.21 c	6.87 ± 0.99 c	11.65 ± 0.96 e
XY508	N0	20.42 ± 2.89 b	60.27 ± 0.73 a	8.94 ± 0.89 b	32.03 ± 0.93 a
	N1	-	23.63 ± 2.08 c	-	25.99 ± 0.57 c
	N2	8.28 ± 0.20 c	20.68 ± 0.61 c	7.03 ± 1.52 c	12.50 ± 1.28 e
12 d after N treatments					
ZH311	N0	33.37 ± 0.57 a	91.05 ± 0.34 a	25.63 ± 0.31 a	32.06 ± 0.97 b
	N1	-	34.20 ± 0.81 b	-	27.75 ± 0.64 c
	N2	9.67 ± 1.78 c	24.92 ± 1.38 c	22.72 ± 0.58 ab	12.41 ± 0.56 d
XY508	N0	24.21 ± 2.95 b	89.29 ± 0.25 a	20.57 ± 0.91 bc	42.50 ± 1.64 a
	N1	-	33.82 ± 0.75 b	-	27.17 ± 0.49 c
	N2	9.23 ± 0.11 c	24.19 ± 0.81 c	18.46 ± 0.53 c	13.63 ± 0.08 d

Note: N0, 0.04 mM; N1, 0.4 mM; N2, 4 mM.

Values with different lowercase letters are significantly different at p < 0.05

within cultivars, values with different uppercase letters are significantly different at p < 0.05 according to the least significant difference test.

### Starch content

**Fig 5:**
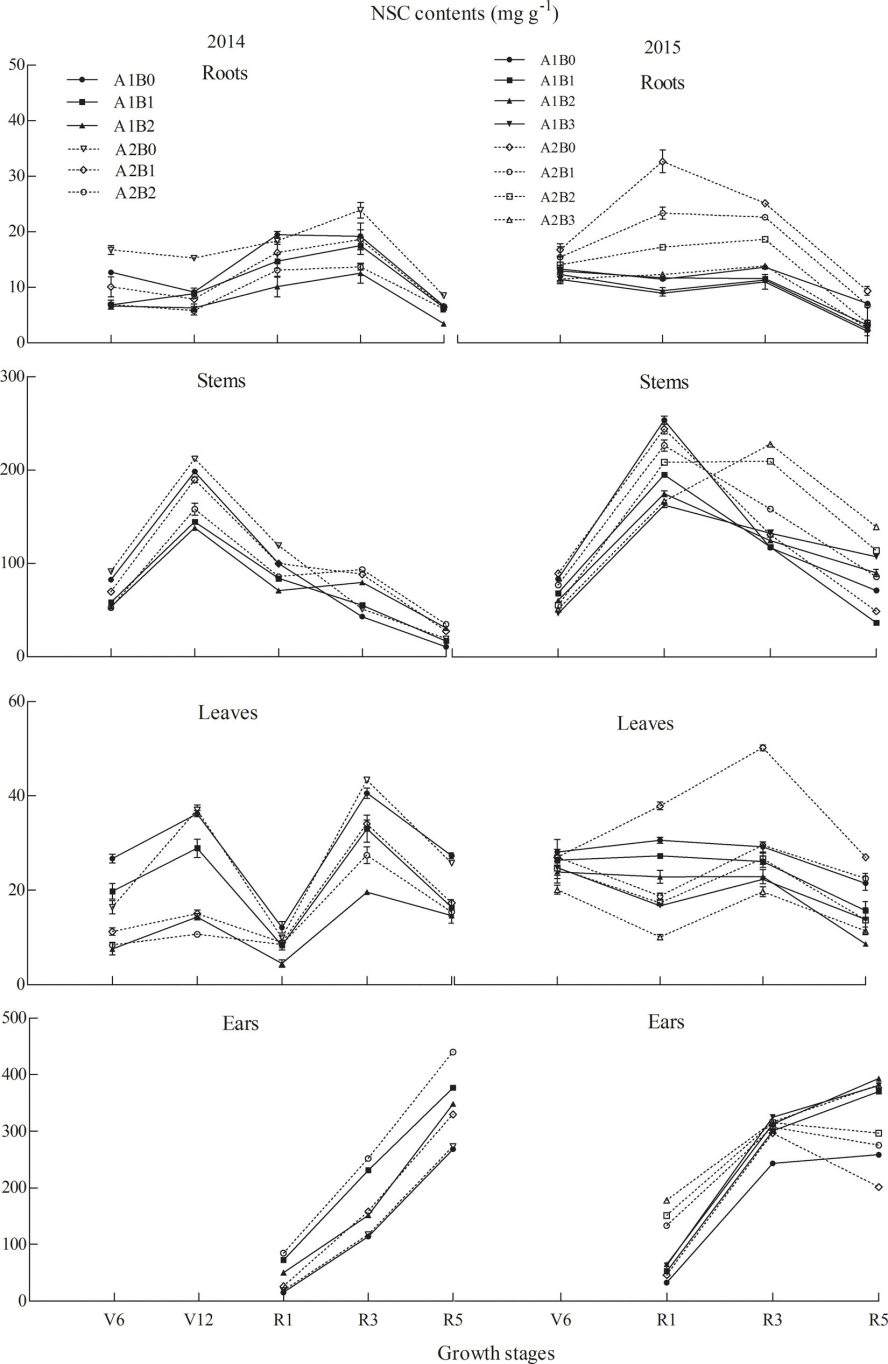
Starch content in different growth periods of maize under various treatments. Note: V6, jointing (V6); V12, big trumpet; R1, silking (R1); R3, milk-ripe; and R5, maturity. A stands for cultivars: A1, ZH311; A2, XY508. B stands for N treatments: B0, 0 kg·ha^-1^; B1, 150 kg·ha^-1^; B2, 300 kg·ha^-1^; and B3, 450 kg·ha^-1^.The point in R5 indicates soluble sugar content in grains.

**Fig 6:**
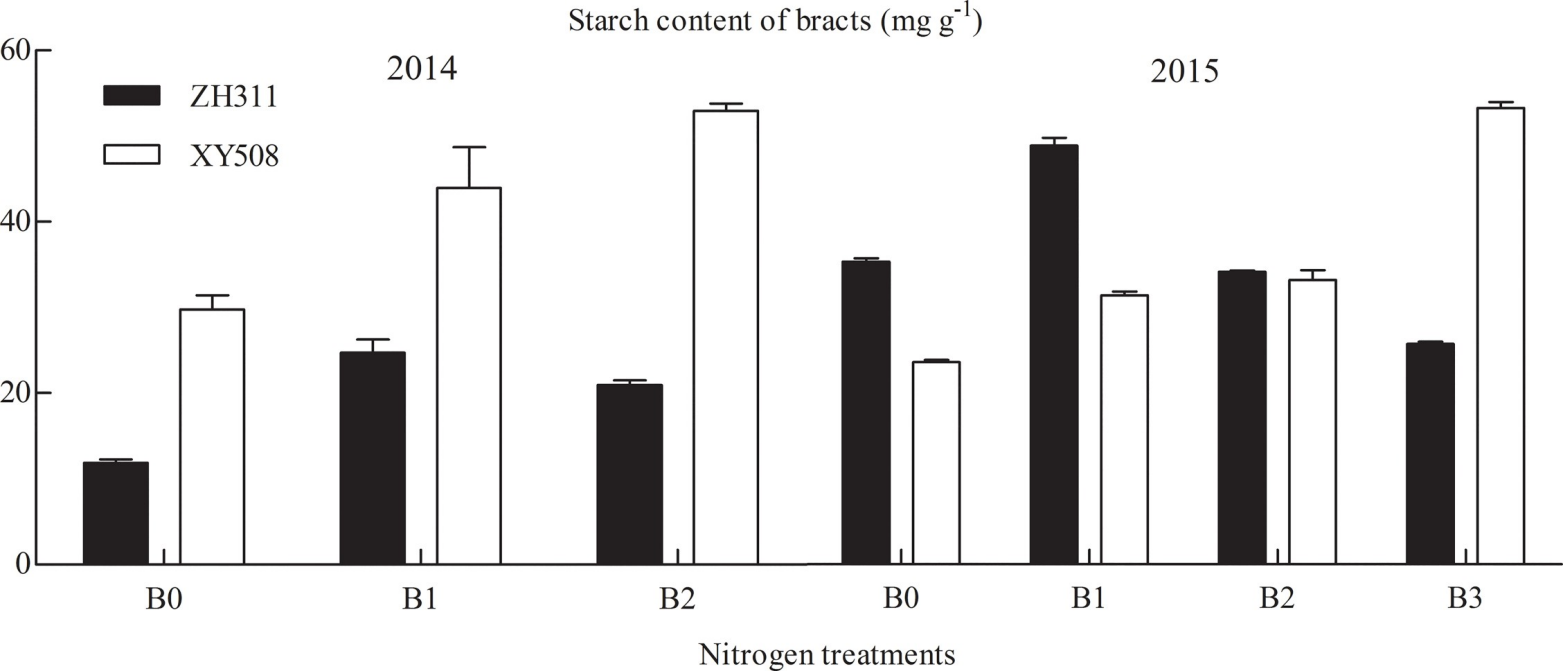
Starch content in the bracts at maturity under different nitrogen treatments. Note: B0, 0 kg·ha^-1^; B1, 150 kg·ha^-1^; B2, 300 kg·ha^-1^; B3, 450 kg·ha^-1^.

In Exp. I (Figs 5 and 6), root starch content in ZH311 decreased by 21.82% under the 150 and 300 kg·ha^-1^ treatments on an average during the two-year growth period compared with that in plants under the 0 kg·ha^-1^ treatment, and the 450 kg·ha^-1^ treatment caused a decrease of 28.05% in 2015. In XY508, the starch content decreased by an average of 38.18% over two years. In 2015, the 450 kg·ha^-1^ treatment caused a decrease of 4.18%. In the leaves, throughout the growth period of ZH311, the starch content under the 150 and 300 kg·ha^-1^ treatments was 41.69% lower than that of plants under the 0 kg·ha^-1^ treatment. In 2015, the 450 kg·ha^-1^ treatment caused a decrease of 50.43%. In XY508, the starch content in the leaves decreased by an average of 30.62% compared with that under the 0 kg·ha^-1^ treatment for two years, and the 450 kg·ha^-1^ treatment caused a decrease of 60.37% in 2015.

During the V6 to R1 stages, under the 150–450 kg·ha^-1^ treatments, the starch content in ZH311 decreased by 21.55%, and during the R1 to R5 stages it increased by 33.79%. The starch content in XY508 during the V6 to R1 stages decreased by 19.63% and increased by 3.82% during the R1 to R5 stages. In the R1 stage, the starch content in the stem of ZH311 decreased by 10.95% (150 kg·ha^-1^) and 23.17% (300 kg·ha^-1^) compared with that under the 0 kg·ha^-1^ treatment for two years, and decreased by 27.85% in 2015 (450 kg·ha^-1^). The starch content in XY508 decreased by 17.10% (150 kg·ha^-1^) and 28.17% (300 kg·ha^-1^), and 36.61% in 2015 (450 kg·ha^-1^). During the R1 and R5 stages, the starch content in the ears of ZH311 under 150–450 kg·ha^-1^ treatments increased by 47.86% on an average over two years compared with that under the 0 kg·ha^-1^ treatment. The starch content in the grain at R5 increased by 31.21% and that in bract increased by 21.71%, and the amplitude was the largest under the 300 kg·ha^-1^ treatment. The starch content in XY508 under the 150–450 kg·ha^-1^ treatments increased by 40.78%, and that in grain increased by 32.52% and in bract increased by 37.22% at R5. The 450 kg·ha^-1^ treatment had the highest effect.

In Exp. II (Table 3), the starch content in ZH311 was averaged at 9 and 12 d for two years. Compared with 0.04 mM, the starch content in the aboveground material under the 4 mM treatment decreased by 45.84%, whereas that in the underground material increased slightly. In 2015, the starch content in the aboveground material under the 0.4 mM treatment decreased by 6.58% and that in the underground material decreased by 9.50%. The starch content in the aboveground material of XY508 decreased on an average by 33.05% (4 mM) at 9 and 12 d for two years, and that in the underground material decreased by 45.81% (4 mM). In 2015, the 0.4 mM treatment decreased the starch content in the aboveground material by 17.92% and that in the underground material by 30.21%.

**Table 3:** Starch content in maize at the seedling stage under different nitrogen treatments (mg g^-1^).

Cultivar	Treatment	Above-ground		Under-ground	
		2016	2017	2016	2017
9 d after N treatments					
ZH311	N0	8.58 ± 2.09 a	17.22 ± 0.34 a	5.75 ± 1.44 a	18.41 ± 0.67 b
	N1	-	14.45 ± 1.98 b	-	16.29 ± 0.14 c
	N2	6.43 ± 0.45 ab	10.61 ± 0.17 c	4.51 ± 0.84 a	12.70 ± 0.06 e
XY508	N0	6.49 ± 1.13 ab	17.44 ± 0.33 a	6.29 ± 0.14 a	20.61 ± 0.17 a
	N1	-	11.46 ± 0.63 c	-	14.68 ± 0.97 d
	N2	5.41 ± 0.64 b	10.52 ± 0.54 c	5.19 ± 0.62 a	11.23 ± 0.64 f
12 d after N treatments					
ZH311	N0	12.98 ± 0.29 a	17.39 ± 0.83 ab	7.64 ± 0.68c	22.64 ± 0.47 a
	N1	-	17.90 ± 0.31 ab	-	20.95 ± 0.92 b
	N2	8.63 ± 0.60 c	17.05 ± 0.85 ab	5.41 ± 0.29 d	12.08 ± 0.81 d
XY508	N0	11.29 ± 1.68 b	18.80 ± 0.26 a	10.44 ± 0.68 a	21.97 ± 0.61 a
	N1	-	18.52 ± 0.77 ab	-	15.02 ± 0.69 c
	N2	10.44 ± 0.24 b	16.80 ± 0.61 b	9.42 ± 0.72 b	11.54 ± 0.23 d

Note: N0, 0.04 mM; N1, 0.4 mM; N2, 4 mM.

Values with different lowercase letters are significantly different at p < 0.05

within cultivars, values with different uppercase letters are significantly different at p < 0.05 according to the least significant difference test.

### Non-structural carbohydrates

**Fig 7:**
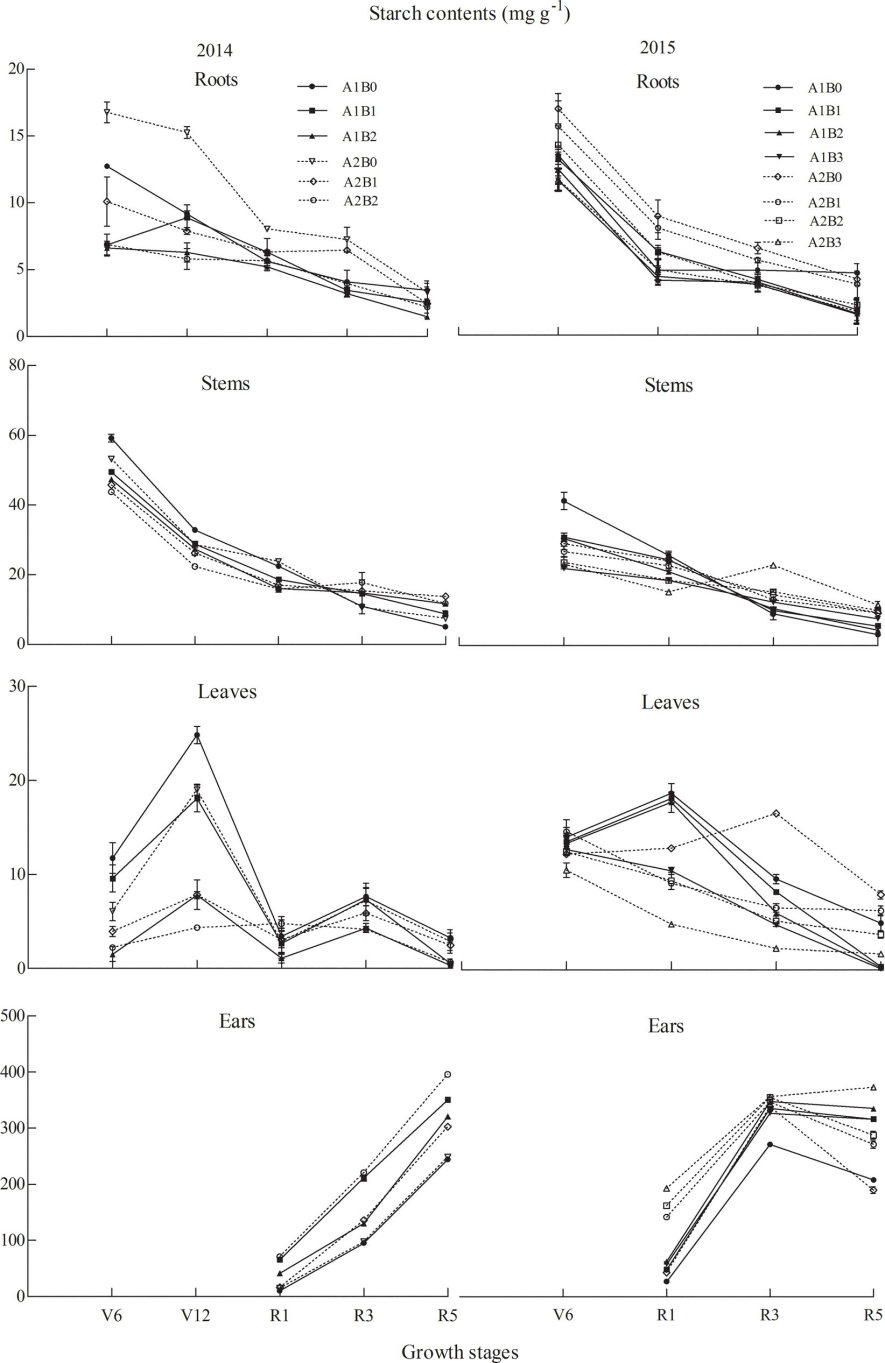
NSC content in different growth periods of maize under various treatments. Note: V6, jointing (V6); V12, big trumpet; R1, silking (R1); R3, milk-ripe; and R5, maturity. A stands for cultivars: A1, ZH311; A2, XY508. B stands for N treatments: B0, 0 kg·ha^-1^; B1, 150 kg·ha^-1^; B2, 300 kg·ha^-1^; and B3, 450 kg·ha^-1^.The point in R5 indicates soluble sugar content in grains.

**Fig 8:**
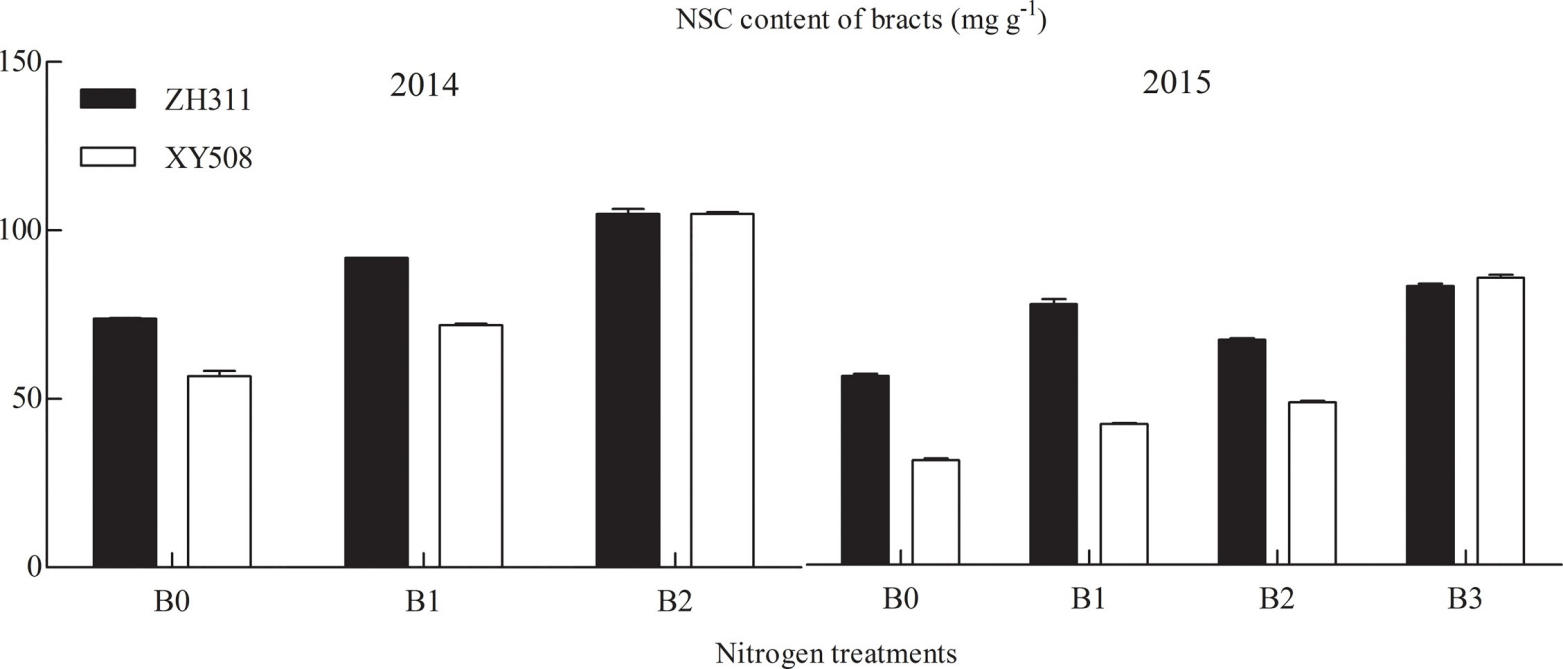
NSC contents of bracts under different nitrogen treatments. Note: B0-0 kg·ha^−1^; B1-150 kg·ha^−1^; B2-300 kg·ha^−1^; B3- 450 kg·ha^−1^.

In Exp. I (Figs 7 and 8), the NSC content in ZH311 roots under the 150–450 kg·ha^-1^ treatments decreased by 27.37% in two years compared with that in those under the 0 kg·ha^-1^ treatment, and in XY508 roots, the 150–450 kg·ha^-1^ treatments caused a decrease of 36.35%. In the leaves, the NSC content in ZH311 under the 150–450 kg·ha^-1^ treatments throughout the growth period of two years was 33.65% lower than that of plants under the 0 kg·ha^-1^ treatment. In the leaves of XY508 under the 150–450 kg·ha^-1^ treatments, the NSC content was 38.51% lower than in those under the 0 kg·ha^-1^ treatment.

In the V6 to R1 stages, the NSC content in ZH311 under the 150–450 kg·ha^-1^ treatments decreased by 28.92%, and in the R1 to R5 stages, it increased by 19.87% on an average for two years. In the same stage, the NSC content in XY508 decreased by 24.59% and in the R1 to R5 stages, it increased by 40.31%. In the R1 stage, the NSC content in the stem of ZH311 decreased by 24.78% on an average in two years under the 150 and 300 kg·ha^-1^ treatments compared with that under the 0 kg·ha^-1^ treatment, but it decreased by 35.83% under the 450 kg·ha^-1^ treatment in 2015. The NSC content in XY508 under the 150–300 kg·ha^-1^ treatments decreased by 16.52% and decreased by 31.73% in 2015 under the 450 kg·ha^-1^ treatment. In the R1 and R3 stages, the NSC content in the ears of ZH311 under the 150–450 kg·ha^-1^ treatments increased by 45% on an average for two years; the NSC content in the grain increased by 29.02% and bract increased by 24.94% in the R5 stage. The NSC content in the ears of XY508 increased by 42.11% in the R1 to R3 stages and that in the grain increased by 31.44% and bract by 38.30% in the R5 stage.

In Exp. II, at 9 d after treatment, the NSC content in the aboveground material of ZH311 under the 0.4 and 4 mM treatments decreased by 52.05% on an average over two years compared with that under the 0.04 mM treatment (Table 4). Whereas, the NSC content in the underground material decreased by 28.58%. At 12 d, the NSC content in the aboveground material decreased by 56.43% and that in the underground material decreased by 26.60%. In XY508 at 9 d, the NSC content in the aboveground material decreased by 54.67% and that in the underground material decreased by 29.48%. The NSC content in the aboveground material decreased by 52.46% and that in the underground material decreased by 34.95% at 12 d, compared with 0.04 mM.

**Table 4:** NSC in maize at the seedling stage (mg g^-1^).

Cultivar	Treatment	Above-ground		Under-ground	
		2016	2017	2016	2017
9 d after N treatments					
ZH311	N0	34.88 ± 0.68 a	80.41 ± 0.26 a	16.96 ± 0.46 a	47.22 ± 0.23 b
	N1	-	45.06 ± 0.58 b	-	36.64 ± 0.85 d
	N2	14.03 ± 1.78 c	31.74 ± 2.24 cd	11.38 ± 0.57 d	24.35 ± 0.15 e
XY508	N0	26.91 ± 0.11 b	77.71 ± 0.36 a	15.23 ± 0.29 b	52.64 ± 0.85 a
	N1	-	35.09 ± 0.97 c	-	40.67 ± 0.53 c
	N2	13.69 ± 0.36 d	31.20 ± 1.52 d	12.22 ± 0.34 c	23.73 ± 1.64 e
12 d after N treatments					
ZH311	N0	46.36 ± 0.29 a	108.44 ± 0.33 a	33.27 ± 1.11 a	54.70 ± 0.38 b
	N1	-	52.09 ± 0.64 b	-	48.70 ± 0.07 c
	N2	18.31 ± 0.12 d	41.97 ± 2.23 c	28.14 ± 0.19 c	24.48 ± 0.76 e
XY508	N0	35.50 ± 2.05 b	108.09 ± 0.47 a	31.01 ± 2.70 b	64.47 ± 0.61 a
	N1	-	52.35 ± 0.53 b	-	42.19 ± 0.67 d
	N2	19.67 ± 0.80 c	40.99 ± 0.47 c	27.88 ± 0.12 c	25.17 ± 0.45 e

Note: N0, 0.04 mM; N1, 0.4 mM; N2, 4 mM.

Values with different lowercase letters are significantly different at p < 0.05

within cultivars, values with different uppercase letters are significantly different at p < 0.05 according to the least significant difference test.

The ratio of SS to starch under the 0–450 kg·ha^-1^ treatments in ZH311 roots on an average during the two-year full growth period was 1.19, 1.11, 1.00, and 0.84, respectively; the stem ratio was 6.62, 4.16, 6.09, and 7.77, respectively; the leaf ratio was 4.99, 19.88, 20.94, and 32.72, respectively; the ear ratio was 0.57, 0.33, 0.38, and 0.29, respectively; the XY508 root ratio was 1.37, 1.24, 1.34, and 1.21, respectively; the stem ratio was 9.07, 10.43, 12.43, and 7.63, respectively; the leaf ratio was 5.56, 5.17, 8.75, and 3.97, respectively; and the ear ratio was 0.36, 0.41, 0.27, and 0.11, respectively. Compared with that in XY508, the SS to starch ratio of ZH311 (root and stem) decreased with the increase in the N application rate, and the ratio under the medium and high N levels was significantly different to that under low N level, while the ratio in the leaves increased.

### Non-structural carbohydrate translocation characteristics in maize with different N tolerance

N application significantly decreased the AR_NSC_ and AC_NSC_ and increased yield (Table 5). With increasing N fertilizer level, the AR_NSC_ gradually decreased, and there was a linear negative correlation between them. The AC_NSC_ also decreased but the relationship between them was explained by an exponential function. For the ATM_NSC_, AR_NSC_, and AC_NSC_, the degree of influence by N fertilizer level was significantly different between the two cultivars. Overall, ZH311 was significantly less affected than XY508 (Table 5). The regression coefficient of AR_NSC_ and N fertilizer levels of ZH311 was 0.0008, while that of XY508 was 0.011. The translocation of NSCs in ZH311 was less affected by N fertilizer levels.

**Table 5:** Apparent transferred mass, apparent ratio of transferred, and apparent contribution of transferred NSC from the stems to grains under different nitrogen treatments.

Cultivar	Treatment	ATM_NSC_ (mg g^-1^)		AR_NSC_ (%)		Yield (t hm^-2^)		AC_NSC_ (%)	
		2014	2015	2014	2015	2014	2015	2014	2015
ZH311	B0	89.48 b	180.68 b	89.49 a	71.99 b	2.06 d	1.52 e	22.80 b	62.41 b
	B1	67.05 d	157.09 c	79.85 b	81.29 a	6.89 b	6.24 bc	5.11 d	13. 22 c
	B2	39.95 f	83.99 f	56.32 f	48.51 d	7.99 a	7.11 a	2.63 f	6.20 e
	B3	**-**	54.85 g	-	34.05 f	-	6.43 b	-	4.48 f
XY508	B0	93.62 a	193.21 a	78.55 c	79.95 a	1.95 d	1.42 e	25.20 a	71.43 a
	B1	74.58 c	139.33 d	74.66 d	62.17 c	6.00 c	5.53 d	6.52 c	13.23 c
	B2	55.58 e	93.30 e	64.69 e	45.29 e	6.86 b	6.10 c	4.25 e	8.03 d
	B3	-	27.21 h	-	16.49 g	**-**	6.04 c	-	2.37 g

Note: N0, 0.04 mM; N1, 0.4 mM; N2, 4 mM.

Values with different lowercase letters are significantly different at p < 0.05

within cultivars, values with different uppercase letters are significantly different at p < 0.05 according to the least significant difference test.

## Discussion

### Non-structural carbohydrates in maize are affected by nitrogen addition

NSC is produced during photosynthesis and acts as a substrate for respiration. It is an important assimilate in the yield of cereal crops and reflects the balance between plant carbon acquisition and expenditure [16]. Its content and distribution affect plant growth and environmental adaptation strategies [23]. N metabolism requires carbon metabolism to provide carbon sources and energy, whereas carbon metabolism requires N metabolism to provide enzymes and photosynthetic pigments. The application of N fertilizer has a significant effect on crop NSC content. Li and Cui [24] showed that a reduction in N fertilizer application is beneficial to increase the activity of sucrose phosphate synthase in rice leaves, thereby increasing plant NSC accumulation and promoting yield. It has been reported that increasing the application of N fertilizer within a certain range can significantly improve the photosynthetic capacity of the leaves and further increase the NSC content, but excessive N fertilizer decreases its content [19]. In our study, we found that N application significantly decreased the NSC content in the vegetative organs of maize. The reasons for this may be: (1) plants use more carbohydrates under medium and high N conditions for growth [20], whereas under low N conditions, plants tend to be smaller and so fewer carbohydrates are required for growth and therefore accumulate in the plant tissues; (2) the inorganic N absorbed by crops from the soil needs photosynthesis to provide a carbon skeleton to be converted into organic N [25], therefore, when the plants grow under higher N conditions, they use more carbon to meet the needs of N metabolism and therefore the NSC levels decrease [26]; (3) some studies have showed that moderate to low N stress increased the NSC content and transport in plants mainly owing to improved starch–sucrose synthesis and transport-related enzyme gene expression and activity [25,27].

In our study, the SS content in the roots increased during the R1 to R3 stages, whereas the starch content decreased, and the total NSC content remained unchanged. This indicated that SS produced by the roots was converted from starch to enhance the metabolic activities of the roots for plant growth consumption [24]. In the field experiment, SS in the roots was not detected before the R1 stage, but it was observed under hydroponic conditions. This may be caused by the material synthesis and transportation in plants under different environments [28]. During the synthesis and transport of carbohydrates, the leaves and stems bear the role of “source” and “sink” throughout the growth stage [15]. The stems act as “sink” organs before silking, and the energy is needed for their growth and development. The carbon source of NSC is derived from the SS transported by the leaves [29], and the other part is derived from the photosynthetic assimilation of non-photosynthetic tissue. After the R1 stage, the grains in the filling stage become the main “sink” organ of the plant. At this time, the function of the stem also changes from "sink" to "source", and it begins to export assimilates [30]. Low-N stress promoted the accumulation of NSC in the stem before vegetative growth (V6–R1 stages) and increased the outward transport of NSC in the stem and sheath during reproductive growth (R1–R5 stages). The assimilation and absorption of N in maize are closely related to its photosynthesis. The leaf is the main photosynthetic sites of plants, and the synthesis and transport of photosynthates are greatly affected by the level of N fertilizer [31]. Our results (Figs 3 and 5) showed that the SS and starch content in the leaves was greatly affected by the growth period. Further research is needed on the variation of leaf NSC response to N and its relationship with the source–sink functions of other organs [32]. In contrast with that in other organs, the NSC content in the ears increased gradually with growth and N application, and the starch content was higher than the SS content [26,33]. Increasing N fertilizer decreased the NSC content in vegetative organs, but increased the grain yield and quality of maize (increased starch content) (Fig 5, Table 5).

The response of NSC content to changing N level was different in different organs, and there were differences among maize cultivars (Figs 3–8, Tables 2–5). Compared with that in XY508, the difference in the SS and NSC content in the organs of ZH311 under N application (150–450 kg ha^−1^) was lower than that under 0 kg ha^-1^ treatment, but the difference in starch content was high (except in the ears). In our study, the ratio of SS to starch in the root and stem of ZH311 decreased with the increase in N application, and the ratio of SS to starch at medium and high N levels was significantly different from that at low N level. The ratio of SS to starch in the root and stem of XY508 increased with the increase in N application.

The accumulation of sucrose in the leaf may inhibit the activity of phosphate synthase, which may lead to the increase in fructose-6-phosphate and thereby inhibit the fructose-1,6-diphosphatase activity [34], increase the content of propionate phosphate in cells, and then decrease the photosynthetic capacity of plants [17]. In this study, the SS to starch ratio in ZH311 leaf increased with the increase in N application. As a N-efficient cultivar, ZH311 could balance and regulate gene expression and activity of sucrose-starch metabolic enzymes in vivo (mainly by improving sugar and starch synthase and invertase activities) [25], and convert stored starch into SS in the root and stem to maintain the balance of cell osmotic pressure to adapt to low N environments. At the same time, the accumulation of SS in the leaf was reduced to maintain higher photosynthetic efficiency [17]. This may be one of the physiological mechanisms of ZH311 tolerance to low N. It is worth noting that the starch content in ZH311 ears and dry matter differ less than those in XY508 ears under different N levels. Previous studies have shown that ZH311 is a highly efficient N absorption variety [21]. To ensure efficient uptake of N before and after flowering, further promoting the utilization and translocation of N in vegetative organs is key to achieve high-yield and N-efficient synergy [26]. That is, efficient uptake of N is the basis and efficient utilization of N is the goal. As the main intermediate between maize photosynthesis, growth, and utilization, NSC storage capacity reflects N uptake and utilization by plants. Therefore, ZH311 promotes the conversion of starch into SS in vegetative organs and maintains high N uptake and utilization efficiency to maintain high dry matter accumulation at different stages of plant development to adapt to low N environments but reduces the effect of low-N stress on starch quantity in maize grains [26,30].

### Effects of nitrogen fertilizer on non-structural carbohydrate translocation in the stems and its contribution to yield

In the R3 stage, the supply of carbon assimilates for grain growth is mainly from the following two sources: photosynthetic assimilates produced after anthesis and photosynthetic assimilates stored in vegetative organs [17,35]. As an important source of grain filling, NSCs stored in the stem before and after the R1 stage provide material for grain yield [18]. Organic matter produced by pre-anthesis photosynthesis is temporarily stored in the stem as starch, whereas assimilates from post-anthesis stem sheaths are transported over long distances as sucrose and distributed to the grains [18,20]. Many researchers have observed that the plant had high N but less starch stored in the stem under high N condition, whereas under medium and low N conditions, the N content in the plant was lower, and the NSC content in the stem increased [15,23,36–37]. The reason for this may be that high N treatment leads to sink competition or source limitation or that under high N conditions, plant respiration increases, and therefore, more carbon is used for active N metabolism and converted into proteins or amino acids that are difficult to release [38]. Under low N conditions, plant growth is significantly inhibited, and the assimilates used for plant growth and development are reduced [39]. Some assimilates are stored in the stem sheaths in the form of NSCs, which is conducive for the persistence of photosynthesis [17,35]. NSCs are transported to grains after grain filling initiation to compensate for insufficient accumulation of grain-filling assimilates caused by reduced photosynthesis.

Our study results (Table 5) revealed that apparent ATM_NSC,_ AR_NSC_ of NSC, and AC_NSC_ of maize stem after the R1 stage were significantly higher under low N conditions than under medium and high N conditions. The difference between AR_NSC_ and AC_NSC_ under high N conditions (450 kg ha^-1^) and non-N treatment was greater in XY508 than in ZH311. The results showed that high N was not conducive for the accumulation and re-transport of NSCs in the stems, resulting in an increase in residual NSCs in the stems and a decrease in the apparent contribution rate of NSCs to yield. In the stem, amylohydrolase, glucosidase, starch phosphatase, and sucrose synthase jointly control the process of starch conversion to sucrose for NSC transport to the grains [25]. Compared with that of ZH311, the effect of N fertilizer application on NSC transport in the stems and sheaths of XY508 was high. Under low N conditions, ZH311 could convert more starch into SS in the stem sheaths after anthesis. This phenomenon may be closely related to the enhancement of sucrose metabolism in ZH311. Sucrose could be effectively converted into glucose and fructose under the action of sucrose synthase and invertase [19]. At the same time, sucrose can be used as a signal molecule in grains, which can induce the expression of sucrose transporter and promote assimilate transport to the ears owing to the decrease in sucrose content. However, our study did not clarify the relationship between carbohydrate accumulation and translocation, and elucidate photosynthate production in the leaves; further study is required in this regard.

## Conclusions

Low-N stress significantly increased the accumulation of NSCs in maize vegetative organs and increased the translocation rate of NSCs in the stem and their apparent contribution to yield, thereby reducing yield loss caused by low-N stress. N application had a greater effect on starch content in the vegetative organs of N-efficient cultivar ZH311, but less effect on the SS and NSC content in the whole plant and starch content in the ears. Compared with that in XY508, as a N-efficient cultivar, ZH311 could convert more starch into SS in the root and stem under low N conditions and reduce the conversion of SS in the leaf to improve photosynthesis to adapt to low N environments, while ensuring that grain yield and starch quantity were not affected. This is evidently an important physiological mechanism involved in this cultivar’s tolerance to low N conditions.
